# Supplementary material for: The causal relationship between the human gut microbiota and pyogenic arthritis: a Mendelian randomization study
Source: Front Cell Infect Microbiol. 2024 Nov 26;14:1452480. doi: 10.3389/fcimb.2024.1452480 (PMC11629706; doi:10.3389/fcimb.2024.1452480)
Supplement: Supplementary file 1 [file DataSheet1.zip › mendelian test/ebi-a-GCST90027852.csv_forest.pdf]

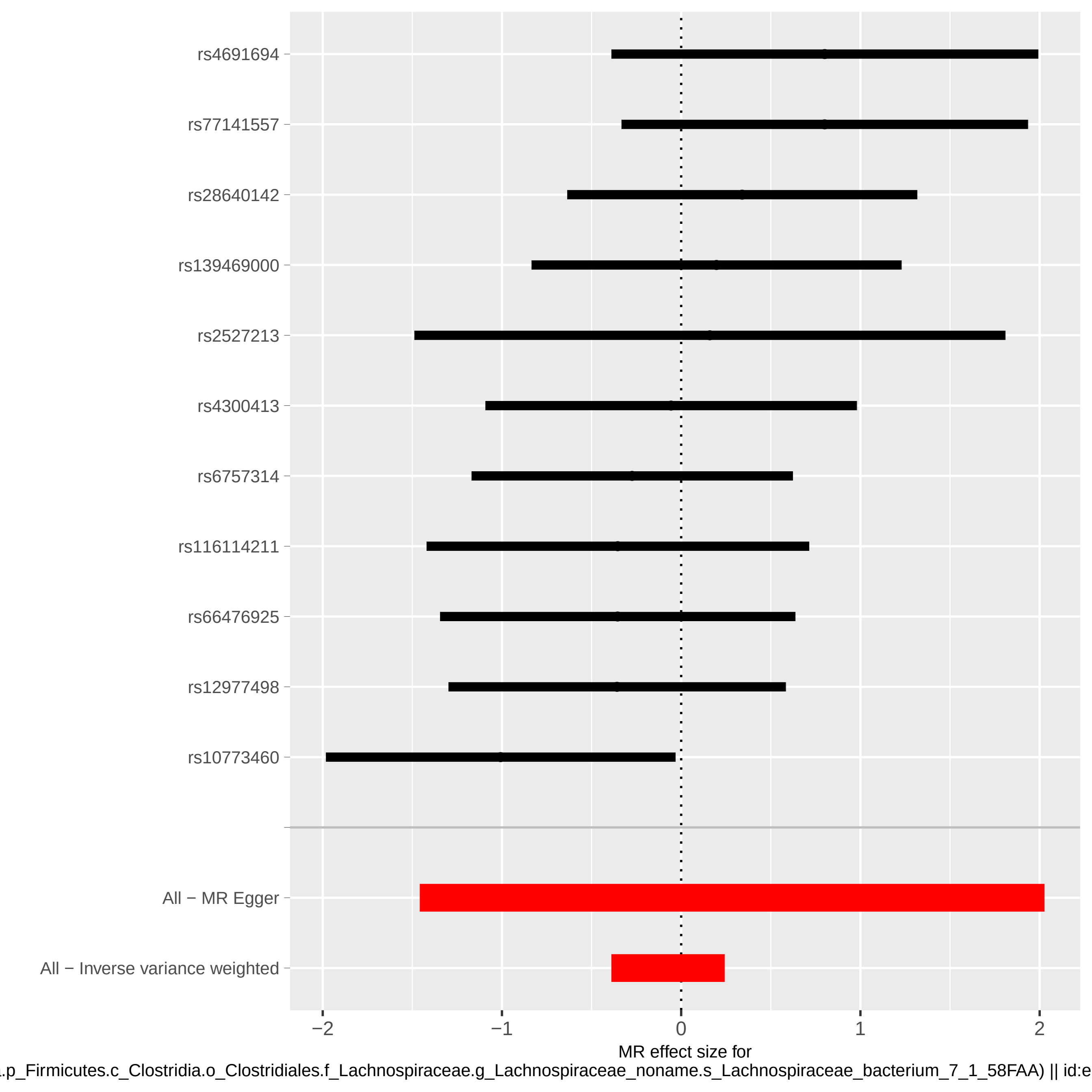
rs4691694
rs77141557
rs28640142
rs139469000
rs2527213
rs4300413
rs6757314
rs116114211
rs66476925
rs12977498
rs10773460
All − MR Egger
All − Inverse variance weighted
−2
−1
0
1
2
MR effect size for
.p_Firmicutes.c_Clostridia.o_Clostridiales.f_Lachnospiraceae.g_Lachnospiraceae_noname.s_Lachnospiraceae_bacterium_7_1_58FAA) || id:e
